# Duplication of 7q34 is specific to juvenile pilocytic astrocytomas and a hallmark of cerebellar and optic pathway tumours

**DOI:** 10.1038/sj.bjc.6605179

**Published:** 2009-07-14

**Authors:** K Jacob, S Albrecht, C Sollier, D Faury, E Sader, A Montpetit, D Serre, P Hauser, M Garami, L Bognar, Z Hanzely, J L Montes, J Atkinson, J-P Farmer, E Bouffet, C Hawkins, U Tabori, N Jabado

**Affiliations:** 1 Department of Pediatrics and Human Genetics, Montreal Children's Hospital, McGill University Health Center, Montreal, Canada; 2 Department of Pathology, Montreal Children's Hospital, McGill University Health Center, Montreal, Canada; 3 McGill University and Genome Quebec Innovation Centre, Montreal, Canada; 4 Second Department of Pediatrics, Faculty of Medicine, Semmelweis University, Budapest, Hungary; 5 Department of Neurosurgery, Medical and Health Science Center, University of Debrecen, Debrecen, Hungary; 6 Division of Neuro-Surgery, Division of Pathology, National Institute of Neurosurgery, Budapest, Hungary; 7 Division of Neurosurgery, Montreal Children's Hospital, McGill University Health Center, Montreal, Canada; 8 Department of Pediatrics, Hospital for Sickkids, Toronto, Canada; 9 Department of Pediatric Neuropathology, Hospital for Sickkids, Toronto, Canada

**Keywords:** SNP arrays, 7q34, JPA, LGA, paediatric, BRAF

## Abstract

**Background:**

Juvenile pilocytic astrocytomas (JPA), a subgroup of low-grade astrocytomas (LGA), are common, heterogeneous and poorly understood subset of brain tumours in children. Chromosomal 7q34 duplication leading to fusion genes formed between *KIAA1549* and *BRAF* and subsequent constitutive activation of BRAF was recently identified in a proportion of LGA, and may be involved in their pathogenesis. Our aim was to investigate additional chromosomal unbalances in LGA and whether incidence of 7q34 duplication is associated with tumour type or location.

**Methods and results:**

Using Illumina-Human-Hap300-Duo and 610-Quad high-resolution-SNP-based arrays and quantitative PCR on genes of interest, we investigated 84 paediatric LGA. We demonstrate that 7q34 duplication is specific to sporadic JPA (35 of 53 – 66%) and does not occur in other LGA subtypes (0 of 27) or NF1-associated-JPA (0 of 4). We also establish that it is site specific as it occurs in the majority of cerebellar JPA (24 of 30 – 80%) followed by brainstem, hypothalamic/optic pathway JPA (10 of 16 – 62.5%) and is rare in hemispheric JPA (1 of 7 – 14%). The MAP-kinase pathway, assessed through ERK phosphorylation, was active in all tumours regardless of 7q34 duplication. Gain of function studies performed on hTERT-immortalised astrocytes show that overexpression of wild-type *BRAF* does not increase cell proliferation or baseline MAPK signalling even if it sensitises cells to EGFR stimulation.

**Conclusions and interpretation:**

Our results suggest that variants of JPA might arise from a unique site-restricted progenitor cell where 7q34 duplication, a hallmark of this tumour-type in association to MAPK-kinase pathway activation, potentially plays a site-specific role in their pathogenesis. Importantly, gain of function abnormalities in components of MAP-Kinase signalling are potentially present in all JPA making this tumour amenable to therapeutic targeting of this pathway.

Juvenile pilocytic astrocytomas (JPA) account for 60–80% of paediatric low-grade astrocytomas (LGA), the most common paediatric brain tumour, and thus are the most frequently encountered subtype of brain neoplasm in children under the age of 19 years. They are classified according to the World Health Organisation (WHO) as WHO grade I (Louis *et al*, 2007), and occur sporadically throughout childhood or arise in up to 15–40% of children affected with neurofibromatosis type 1 (NF1; Pollack and Mulvihill, 1997). JPA exhibit distinct features, readily distinguishable from their other LGA counterparts, including clinical course and molecular characteristics, and have better prognosis in affected children (Gajjar *et al*, 1997; Ishii *et al*, 1998; Cheng *et al*, 2000; Tada *et al*, 2003; Broniscer *et al*, 2007; Fisher *et al*, 2008).

Even though they share similar histology, there is heterogeneity between sporadic JPA in terms of localisation, radiologic features, histologic atypia and clinical behaviour, all of which argue for the possibility of genetic disparity between potential JPA subgroups. Typically, JPA occur as exophytic cerebellar tumours, however, they can also arise in the brain stem or the optic pathway, where they behave more aggressively than NF1-associated JPA (Grill *et al*, 2000), or, more rarely, in the cerebral hemispheres. Maximal surgical resection is the mainstay of therapy, and failure to achieve it remains the main therapeutic concern. Although cerebellar JPA are readily amenable to complete surgical resection, in other less anatomically accessible locations, surgery may result in the persistence of residual disease, which can require further therapy for tumour control, and ultimately lead to increased morbidity/mortality. In addition, some of these extracerebellar JPA seem less circumscribed and may exhibit atypical pathologic features, leading to pathologic misdiagnosis, including into higher grade tumours.

Until recently, the few genetic abnormalities documented in JPA mainly identified chromosomal gains of 7q and trisomy of chromosomes 5, 7 or 8 in some tumours (White *et al*, 1995; Rickert and Paulus, 2004; Wemmert *et al*, 2006). Recently several consecutive papers described duplication of 7q34 in LGA including JPA (Bar *et al*, 2008; Deshmukh *et al*, 2008; Jones *et al*, 2008; Pfister *et al*, 2008; Sievert *et al*, 2008). However, the data reported are conflicting regarding the subgroup of LGA affected by this genetic event, the size of the duplication and its anatomical localisation within the brain. Indeed, Deshmukh *et al* (2008) identified amplification of 7q34 in 8 of 10 cerebellar JPA at 138151200–139456000, which included *HIPK2*, a potential gene of interest. The authors further showed using an immunohistochemical approach that overexpression of HIPK2 was more frequent in LGA than in high-grade gliomas, and that it was more common in infratentorial tumours. Their silencing of *HIPK2* in U87 (a glioblastoma (GBM) cell line) decreased the cells proliferation rate. Pfister *et al* (2008) identified duplication of 7q34 within 139186224–140156951 in 30 of 66 (45.5%) paediatric LGA. This region included *BRAF*, a gene which was not in the genetic interval described in Deshmukh *et al* (2008). The authors confirmed the absence of previously described *BRAF* oncogenic mutations in their sample set, and showed that silencing of *BRAF* in a LGA cell line decreased cell proliferation rates. However, based on lower resolution BAC arrays, they did not precisely map the genetic region missing other genes potentially critical in the pathogenesis of LGA, including *HIPK2*. In addition, data from this report suggest that LGA other than JPA may harbour 7q34 duplication and that this genetic alteration is more frequent in non-cerebellar tumours.

Recent reports have indicated a central role for the mitogen-activated protein kinase (MAPK) pathway in the tumorigenesis of pilocytic astrocytomas and showed that duplication at 7q34 leads to a fusion between *KIAA1549* and *BRAF* resulting in constitutive activation of the BRAF kinase (Jones *et al*, 2008; Sievert *et al*, 2008). In particular, Jones *et al* (2008) focused on JPA and describe a tandem duplication at 7q34 producing a transforming BRAF fusion gene in 29 of 44 tumours (66%), and V600E point mutation of BRAF in two further cases. Sievert *et al* indicated that 7q34 duplication occurs in 17 of 22 JPA but also report it in 3 of 6 diffuse astrocytomas (LGA grade II).

To determine the specificity of 7q34 duplication to a given subgroup of LGA and identify additional genetic aberrations in tumours that do not carry this duplication, we investigated 115 paediatric brain tumour samples including 57 JPA, and 27 diffuse astrocytomas (Tables 1, 2a and b). Our data indicate that 7q34 duplication is exclusive to JPA and a hallmark of specific anatomical localisations of these tumours within the brain. We identify additional genetic abnormalities in JPA that do not harbour 7q34 duplication, which may help shed light on their pathogenesis. Data from gain of function analysis in immortalised astrocytes further confirm that increased expression of wild-type *BRAF* is unable to cause malignant transformation on its own however may contribute to an increased response to exogenous triggering of membrane receptors. Moreover, we show that the MAPK pathway is active in all JPA regardless of 7q34 duplication and that all of these tumours may be amenable to therapeutic targeting of this pathway.

## Materials and methods

### Samples

All samples were obtained with informed consent after approval of the Institutional Review Board of the respective hospitals they were treated in, and independently reviewed by senior paediatric neuropathologists (SA, CH, ZH) according to the WHO guidelines (Kleihues *et al*, 2002). A total of 115 paediatric brain tumours (average 9.4±4.7 years) were analysed (Tables 1, 2a and b); 57 JPA (53 sporadic, 4 from NF1 Patients), 27 diffuse astrocytomas (grade II), 1 grade II ependymoma, 5 grade I gangliogliomas and 25 high-grade astrocytomas (HGA) were included. There were no pylomixoid variants within the JPA included within this study. All samples were taken at the time of the first surgery before further treatment, when needed. Tissues were obtained from the London/Ontario Tumor Bank, and from collaborators in Montreal, Toronto and Hungary.

### DNA extraction and hybridisation, SNP analysis

DNA from frozen tumours was extracted as described previously (Wong *et al*, 2006). For SNP analysis, DNA (250 ng) from 40 samples was assayed with the Human Hap300-Duo (*N*=28) and the 610-Qad (*N*=16) genotyping beadchips according to the recommendations of the manufacturer (Illumina, San Diego, CA, USA). These BeadChips enable whole-genome genotyping of respectively over 300 000 and 610 000 tagSNP markers derived from the International HapMap Project (www.hapmap.org) with a mean intermaker distance of 10 and 5 kb respectively. Image intensities were extracted using Illumina's BeadScan software. Data for each BeadChip were self-normalised using information contained within the array. Penn-CNV (Wang *et al*, 2007) and GqCNV (D Serre *et al*, unpublished) algorithms were applied on the genotype data derived from the 40 LGA. Only the alterations that were detected by both algorithm and that contained more than 5 consecutive SNPs were considered in this study. They were further confirmed by visualisation in the BeadStudio.

### Validation of copy number changes by quantitative real-time PCR

Quantitative real-time PCR (q-PCR) was done on an ABI-Prism 7000 sequence detector (Applied Biosystems, Bedford, MA, USA) using a SYBR Green kit (Applied Biosystems). The target locus from each tumour DNA was normalised to the reference, Line-1 as previously described (Wong *et al*, 2006; Supplementary Table 1).

### Cell lines, antibodies and transfections

hTERT-immortalised human astrocytes (kind gift of Dr A Guha, Labbatt Brain Tumour Research Centre, Ontario, Canada) were grown and transfected with 2 *μ*g of plasmid DNA encoding wild-type C-Myc-Braf using Fugene 6 (Roche, Mississauga, ON, Canada) as previously described (Shi *et al*, 2004). Unless stated otherwise, all antibodies used were obtained from Cell Signaling (Danvers, MA, USA). Transfected cells were used at 0, 48 and 72 h posttransfection. HIPK2 plasmids were generously provided by Dr Gabriella D'Orazi (Regina Elena Cancer Institute, Rome, Italy), and BRAF plasmids by Dr Richard Marais (Cancer Research, London, UK).

### Western blot and immunofluorescence analysis

Extracts were prepared from cell pellets and western blot analysis performed on total lysates as previously described (Jabado *et al*, 1998; Rajasekhar *et al*, 2003). Cross-reactivity was visualised by ECL chemiluminescence (Amersham) on a phosphorimager. For immunofluorescence analysis images were acquired using a Retiga 1300 digital camera (QIMAGING) and a Zeiss confocal microscope.

### Immunohistochemical analysis

Immunohistochemical analyses for phospho-Erk (pErk), were performed and the slides scored as previously described (Faury *et al*, 2007).

### [3H]Thymidine incorporation assay

DMEM (10 ml) containing 7.4 kBq (0.2 mCi) of [methyl-3H]thymidine (Amersham Pharmacia Biotech Europe, Freiburg, Germany; Batch 215, 65 Ci mM) were added to each microplate well. Experiments were done in triplicate at least three times with identical results.

## Results

### Copy number variants in 40 LGA

**Figure 1:**
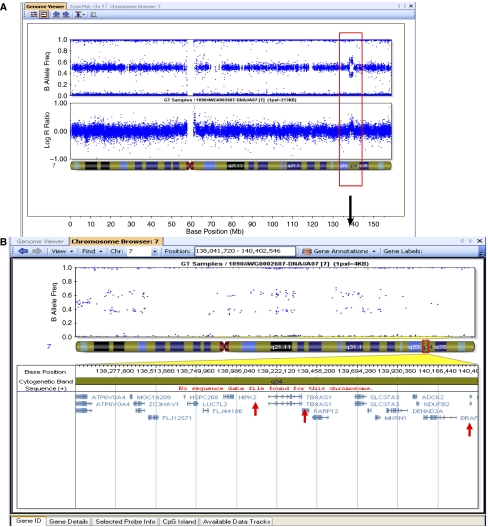

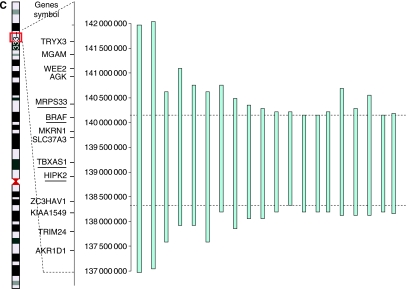
Duplication of 7q34 visualised using BeadStudio. (**A**) Chromosome-wide data showing duplication at 7q34 through the increase in the log R ratio values (top) and split in the B allele frequencies (bottom) plotted for each SNP for one JPA sample (Patient 10; Table 1). (**B**) Zoom-in showing genes included within the region of interest. (**C**) Detailed view of the 7q34 locus amplified in each of the 20 JPA samples. Genes within and outside the region of interest are shown on the left. Genes we further used to validate duplication within this dataset and an additional dataset of 35 tumours are bolded.

To chart genomic alterations in our sample set, we first performed a high-resolution genome-wide screen of the samples using the SNP arrays. The copy number variant (CNV) analysis of the resulting dataset generated on the Human Hap300-Duo and 610-Qad arrays gave similar results for both platforms. All samples had at least one CNV and the overall frequency of CNVs according to the chromosomal position showed that most tumours had only focal abnormalities, some of them previously reported. Most LGA did not have chromosome-wide gains or deletions, with the exception of two JPA samples, which had gains of the whole chromosome 7 (Supplementary Table 2). Regions with loss-of-heterozygosity were rarely found in LGA. We found a single region showing recurrent gain of 7q34 in 20 of 40 (50%) samples (minimal common region of gain for all tumours on chromosome 7:138380901–140119915, NCBI Build 36.3; Figure 1). The gain specifically corresponds to a chromosomal duplication, according to the Illumina Plots. A total copy number of 3 was inferred based of the logR ratio plot that is characterised by an upward deflection from 0 to 0.35 and by a split in the heterozygous allele frequencies (B-allele frequency measure) into two populations, one located at 0.67 (2 : 1 ratio) and the other at 0.33 (1 : 2 ratio; Figure 1). This gain in 7q34 is a somatic event as it was present in the tumour and not in DNA from peripheral blood taken from the same patients (*n*=7), thus excluding a germ-line segmental duplication (data not shown). It was not found in a set of 1363 control DNA analysed with the Illumina Human-Hap 300K platform (Hakonarson *et al*, 2007) or in the 25 HGA included in this study.

### 7q34 duplication involves both BRAF and HIPK2 genes and is more frequent in extrahemispheric JPA

**Figure 2:**
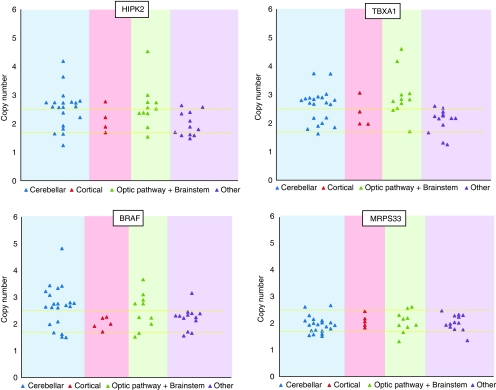
*HIPK2*, *TBXAS1*, *BRAF* and *MRPS33* copy number in the brain tumours included in this study. DNA qPCR-based copy number for *HIPK2*, *TBXAS1*, *BRAF* and *MRPS33* are plotted. The cut-off for DNA copy number was set using the mean of ±2 s.d. (plotted lines) against *MRPS33*, which was located after the genetic interval of interest (Figure 1).

To validate amplification of the genetic interval we identified, DNA was extracted from 17 samples analysed by SNP arrays and an additional independent set of 55 samples including 22 JPA (Tables 1, 2a and b). We performed quantitative real-time PCR (qPCR) on genes included within (*HIPK2* – *homeodomain-interacting protein kinase 2*; *TBXAS1 – thromboxane synthetase 1*), at the edge (*BRAF*) and located just after (*MRPS33*) the interval of interest (Figure 1). The resulting profiles further confirm those obtained by SNP arrays and show that 26 of 72 samples have a detectable amplification of *HIPK2*, *TBXAS1* and *BRAF*, and that most samples have normal copy number of *MRPS33*, which was located just outside of the genetic interval of interest on chromosome 7q34 (Figures 1 and 2; Tables 1, 2a and b). Levels of messenger RNA for *BRAF* and *HIPK2* were also tested using quantitative RT-PCR, as previously described (Faury *et al*, 2007; Haque *et al*, 2007), in seven samples with 7q34 duplication, and were in the range of 1.7–5 (data not shown).

Based on concordant results we considered the amplification of *HIPK2*, *TBXAS1* and *BRAF* by qPCR analysis to reflect duplication of the 7q34 region. We thus combined SNP and qPCR data, and further determined the incidence of 7q34 duplication based on histology, for example, sporadic JPA (*N*=53), NF1-associated JPA (*N*=4), diffuse astrocytomas (*N*=27) and the other paediatric brain tumours (*N*=31). 7q34 duplication was only present in sporadic JPA. Indeed, it was identified in 35 of 53 (66%) JPA, and was absent in NF1-associated JPA and the other brain tumours (Tables 1, 2a and b; Figure 2). Remarkably, this duplication was more prevalent in tumours originating from specific sites within the brain. In this regard, we found this amplification in 24 of 30 (80%) of cerebellar and 10 of 16 (62.5%) of brainstem/hypothalamic/optic-pathway JPA, whereas only 1 of 7 of hemispheric JPA had this duplication, which did not include BRAF (Tables 1, 2a and b; Figure 2; *P*<0.001). We also sequenced *BRAF* in 52 samples and found point mutations affecting the hot spot codon 600 in exon 15 of *BRAF*, V600E (an activating mutation previously described in melanomas and other cancers (Wan *et al*, 2004)) only in 1 JPA and 1 grade II ganglioglioma (Tables 1, 2a and b; Supplementary Table 3) in keeping with previous studies (Jeuken *et al*, 2007; Jones *et al*, 2008; Pfister *et al*, 2008).

### Copy number variants in JPA without 7q34 duplication

We investigated genetic aberrations occurring specifically in JPA not carrying 7q34 duplication analysed by SNP arrays (*N*=10) and identified recurrent abnormalities (Table 3). Amplification of 19p13 including killer receptor inhibitory genes (KIR) genes regulating the activity of natural killer cells within the immune system was identified in 5 of 10 JPA. A recurrent region of amplification at 12p11.21 was also identified in 7 of 10 JPA and 2 of 5 grade II LGA. It includes genes with unknown function and *OVOS2*, a gene similar to ovostatin, a proteinase inhibitor involved in innate immune responses. These data suggest that genes involved in immune modulation may be important in the pathogenesis of another subgroup of JPA, which does not harbour 7q34 duplication.

### The MAPK pathway is activated in all JPA regardless of 7q34 duplication

**Figure 3:**
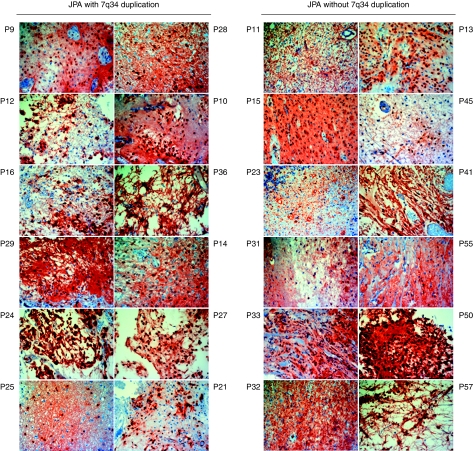
The astrocytic component of all the brain tumours included in this study show MAPK pathway activation regardless of BRAF copy number. Immunohistochemical analyses for phosphorylated ERK (pERK), used as a surrogate marker of MAPK pathway activation were performed for the 52 samples included in this study for which formalin-fixed paraffin-embedded slides were available. Sections were stained using anti-pERK followed by detection using the DAKO kit (red accounts for positive staining) and hematoxylin counterstaining. Staining intensity was scored as in (Faury *et al*, 2007; Haque *et al*, 2007). Full characteristics of samples are provided in Tables 1, 2a and b.

To assess whether BRAF amplification in the absence of mutation can be correlated with increased activity of the RAF/MEK/ERK pathway, we used immunohistochemical analysis based on antibodies against the phosphorylated (activated) form of ERK1–2 (pERK). Sections from samples for which we had fixed paraffin embedded slides were tested for pERK (Tables 1, 2a and b; Figure 3) immunoreactivity and scored as previously described (Faury *et al*, 2007). Results show that the astrocytic component of all JPA stained positively for pERK. This result indicates that the MAPK pathway is triggered in LGA, including JPA, regardless of *BRAF* copy number status or activating mutation.

### Functional characterisation of BRAF and/or HIPK2 overexpression in immortalised mature astrocytes

**Figure 4:**
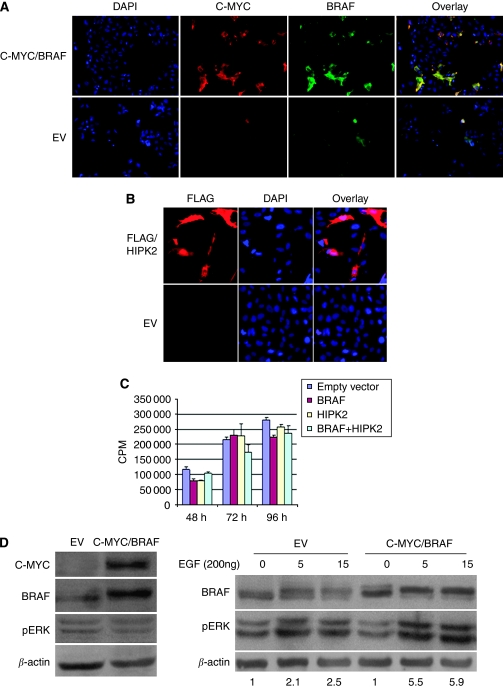
Functional analysis of *BRAF* and *HIPK2* overexpression in hTERT-immortalised astrocytes. (**A**) Immunofluorescence analysis of hTERT-immortalised astrocytes transfected with CMYC/BRAF or the empty vector (EV) at 48 h using an antibody recognising the C-MYC tag (red), BRAF (green) and DAPI counterstaining (blue). (**B**) Immunofluorescence analysis of hTERT-immortalised astrocytes transfected with FLAG/HIPK2 or the EV at 48 h using an antibody recognising the FLAG tag (red) and DAPI counterstaining (blue). (**C**) Proliferation of hTERT-immortalised astrocytes was assessed at 48, 72 and 96 h following transfection with mock, BRAF, HIPK2 or both genes. No difference in the rate of cell growth was observed between the transfectant cells. Results represent the median of three separate experiments performed in triplicates. (**C**) Total cell extracts of hTERT-immortalised astrocytes transfected with the empty-vector or C-Myc-tagged BRAF. Cells were serum starved overnight before protein lysate extraction. (**D**) Left panel: hTERT-immortalised astrocytes were transiently transfected with CMYC/BRAF or the empty vector. Cells were serum starved overnight and total protein lysates extracted at 48 h posttransfection. Western blot analysis for C-Myc, BRAF, phopshoERK (pERK) and *β*-actin (loading control) was performed. Right panel: Empty-vector (EV) and C-Myc/BRAF transfectant hTERT-immortalised astrocytes were stimulated with 200 ng of epidermal growth factor (EGF) for 5 and 15 min. Total cell lysates extracted at baseline (0) and following activation (5, 15 min) with EGF were immunoblotted using antibodies against *β*-actin (loading control) and phosphorylated ERK (pERK), C-Myc and BRAF. Note the shift of BRAF immunoreactive band following EGF activation which indicates phosphorylation of the kinase and increase in its molecular weight. Importantly, increase in pERK levels relative to baseline following EGF stimulation was two fold higher in C-MYC/BRAF transfectant cells than in EV transfectants. Results represent the median of three separate experiments (*P*<0.001).

To assess the potential of increased levels of wild-type BRAF to drive increased activity of mitogenic pathways in cells of glial origin we used hTERT-immortalised astrocytes (Kamnasaran *et al*, 2007) as recipients of transient overexpression of genes from the 7q34 region, wild-type (WT) *BRAF*, *HIPK2* or both. Mutant *V600E BRAF* was used as a positive control for transformation of astrocytes. Transfection efficiency was estimated by immunofluorescence analysis against the C-Myc and FLAG tags to be approximately ∼40–50% at 48, 72 and 96 h (Figure 4A and B). No foci of transformation or morphological changes were observed in cells transfected with WT constructs, whereas transformation foci were readily distinguishable in V600E transfectant cells. Proliferation assays revealed no change in growth of cells overexpressing of *BRAF, HIPK2* or both, compared to their mock-transfected controls (Figure 4C). Similarly, overexpression of BRAF had no discernible effect on the baseline level of ERK expression or phosphorylation (Figure 4D). Indeed, in serum starved EV and C-Myc-BRAF transfectant cells pERK was similar in intensity in both settings, in keeping with previous findings in COS cells (Ciampi *et al*, 2005). Remarkably, exposure of BRAF overexpressing cells to ligands of the epidermal growth factor receptor (EGFR), such as transforming growth factor-*α* or EGF increased pERK levels by a mean of ∼5.9-fold relative to 2.3-fold in empty-vector transfectants (mean of three distinct experiments, Figure 4D). These results indicate that although BRAF overexpression may not be sufficient to drive proliferative responses of astrocytic cells it may sensitise them to exogenous growth factors.

## Discussion

We show that somatic duplication of 7q34 in LGA is specific to JPA. We also establish that its prevalence varies with the site of origin within the brain of the JPA, and is more frequent in cerebellar (24 of 30 – 80%) followed by brainstem and optic pathway tumours (10 of 16 – 62.5%), whereas it is rare in hemispheric JPA (1 of 7 – 14%; Tables 1, 2a and b; Figures 1 and 2). Our analysis and the several validation steps we performed characterise this region with increased precision compared to the recently published concurrent studies, which missed either BRAF or HIPK2 (Deshmukh *et al*, 2008; Jones *et al*, 2008; Pfister *et al*, 2008) and confirm that 7q34 amplification includes both *BRAF* and *HIPK2* in as many as 34 of 35 JPA samples. We identify additional genetic regions in JPA without 7q34 duplication (Table 3) and show that most genes within these regions are modulators of the immune system.

Specificity of 7q34 duplication to JPA and its prevalence in infratentorial tumours contrast with previous reports alluding that 7q34 duplication may be present in non-JPA LGA (Deshmukh *et al*, 2008; Pfister *et al*, 2008; Sievert *et al*, 2008) and is more frequent in non-cerebellar LGA (Pfister *et al*, 2008; Sievert *et al*, 2008). Deshmukh *et al* showed 7q34 duplication in 8 of 10 cerebellar JPA and further used increased HIPK2 expression on tissue microarray as a surrogate for marker for this genetic event. Other molecular events, distinct from 7q34 duplication, may lead to *HIPK2* overexpression (Sombroek and Hofmann, 2009) and may account for increased HIPK2 levels in tumours other than JPA. We chose to validate the genetic interval we identified through concordant increased copy number by qPCR of three consecutive genes within it (*HIPK2*, *TBXAS1*, *BRAF*) in a large number of samples, and thus show specificity of 7q34 duplication to JPA. The lower resolution BAC-arrays used by Pfister *et al* led them to underestimate the size of the duplication (0.94 Mb instead of the ∼1.74 Mb, we and others describe) and might have led the authors to miss this duplication in a number of cerebellar JPA samples. Indeed, when we revisited data collectively published by the other groups we found that JPA within the cerebellum have the highest incidence for this duplication. In addition, in Jones *et al* (2008), which focused on JPA, the authors indicate 7q34 duplication might be more prevalent in infratentorial tumours, also in keeping with our data. They also show that one sample was classified as a grade II astocytoma; however, the child had prolonged disease-free survival more in keeping with a diagnosis of JPA. This misclassification might also account for the findings of 7q34 duplication in some non-JPA LGA in the study by the other groups (Deshmukh *et al*, 2008; Pfister *et al*, 2008; Sievert *et al*, 2008). The central review of samples by senior paediatric neuropathologists we performed has been shown to increase reproducibility of neuropathologic classification of tumours (Pollack *et al*, 2003) and may account for increased accuracy in our dataset. Our findings and these from recent studies alluding to the presence of different molecular signatures in sporadic JPA based on its region of origin (Sharma *et al*, 2007) make it tempting to speculate that, like CNS ependymoma (Taylor *et al*, 2005), variants of JPA might arise from a unique site-restricted progenitor cell.

RAF kinases are components of a conserved signalling pathway that regulates cellular responses to extracellular signals (Wan *et al*, 2004). Their role in glioma formation, and the role of the MAPK cascade they trigger, have been identified in tumour samples and further confirmed in mouse models (Jeuken *et al*, 2007; Pritchard *et al*, 2007; Lyustikman *et al*, 2008; Pfister *et al*, 2008). *NF1* encodes for a Ras-GTPAse and its loss of function may lead to activation of Ras/RAF signalling and coincides with formation of indolent JPA mainly within the optic pathway. Our exploration of immortalised astrocytes show that overexpression of wild-type *BRAF* may not be sufficient by itself to mediate the activation of the MAPK cascade. In keeping with these data, only oncogenic *BRAF* mutations have been implicated in tumours of epithelial (Rajagopalan *et al*, 2002) and neuroectodermal origin (Brose *et al*, 2002; Pollock *et al*, 2003). 7q34 duplication in JPA was recently shown to produce novel oncogenic BRAF fusion genes with transforming capacity (Jones *et al*, 2008), similar to findings in radiation-associated papillary thyroid cancer (Ciampi *et al*, 2005) where an intrachromosomal inversion, and not duplication, let to this oncogenic event. These data indicate that wild-type *BRAF* would be unlikely to induce transformation. Tumours in which the duplication occurs without this in-frame fusion or in patients with trisomy of the full chromosome 7 require other mechanisms to activate BRAF and induce oncogenesis. Intriguingly, our data suggest that all JPA have active MAPK pathway regardless of 7q34 duplication (Figure 3). In keeping with the importance of the MAPK pathway in JPA genesis, two additional alternative mechanisms resulting in MAPK activation were very recently identified in JPA (Jones *et al*, 2009). This group studied the 10 JPA for which V600E point mutation of BRAF (2 of 44), clinical diagnosis of NF1 (3 of 44) or the common BRAF fusion following 7q34 duplication (29 of 44) could not be found (Jones *et al*, 2008). In one patient they found tandem duplication at 3p25 producing an in-frame oncogenic fusion between SRGAP3 and RAF1, a finding corroborated by a concurrent group on additional patients with JPA and no clinical NF1, 7q34 duplication or BRAF mutations (Forshew *et al*, 2009). This genetic event bears striking resemblance to the common *KIAA1549 BRAF* fusion event and the fusion protein includes the Raf1 kinase domain, and shows elevated kinase activity when compared with wild-type Raf1. Secondly, in one patient with JPA a novel 3 bp insertion at codon 598 in BRAF mimics the hotspot V600E mutation to produce a transforming, constitutively active BRaf kinase (Jones *et al*, 2009). These findings further imply that BRAF or RAF1 constitutive activation, achieved through different genetic events, converge to activate the MAPK pathway and that this pathway could be clinically targeted in all JPA.

Gains in 7q34 have been described in other cancers including ovarian cancer. However these are amplifications and not duplications and the region involved has some but no complete overlap with the one identified in JPA. Also, genetic analysis of medulloblastomas (Gilbertson and Ellison, 2008), ependymomas (Taylor *et al*, 2005) and high-grade astrocytomas (our data) do not identify 7q34 duplication in these tumours making this genetic event specific to JPA. Unscheduled activation of the MAPK pathway may lead to cellular senescence, which could function to limit tumour development (Courtois-Cox *et al*, 2006). Therefore we also postulate that oncogenic BRAF may cooperate with other, presently unknown changes, to drive formation of a subset of JPA at the same time it may drive activation of other pathways including cellular senescence. Ultimately, this may make this tumour less aggressive, similar to what is observed in melanocytes where oncogenic *BRAF* mutations result only in naevi formation. As complete surgical removal is the rule for cerebellar JPA, studies compiling higher numbers of JPA, occurring in other locations, are needed to establish whether 7q34 duplication is indeed associated with improved progression-free survival, as well as the biological mechanisms controlling these variables.

In summary, our studies suggest that the 7q34 region may play a site-specific role in pathogenesis of JPA and that the involvement of an active MAP kinase signalling pathway through oncogenic BRAF and other RAF family members in this process is crucial. They further emphasise the relevance of therapeutic targeting of this pathway in all JPA. To avoid misdiagnosis and adapt therapies, all infratentorial LGA should be screened by immunohistochemistry for active MAP kinase pathway (phosphorylated ERK) and further tested for the presence of 7q34 duplication, then, in its absence, 3p25 gain or BRAF mutation. Further functional evaluation of genes included within this genetic interval, others we identify herein, and others involved in the MAP kinase cascade and their correlation in larger sample sets to the site of origin, age of the patient and progression-free survival will help shed light on the pathogenesis of JPA.

## Figures and Tables

**Table 1:** Characteristics of the 53 sporadic juvenile pilocytic astrocytomas (JPA) included in the study

**Patients**	**Age (years)**	**Location**	**HIPK2 (qPCR)**	**BRAF (qPCR)**	**SNP array**	**Amplification of 7q34**	**pERK (IHC)**	**BRAFV600E**	
*Cerebellar JPA (*n*=30)*									
1	6	Cerebellar	ND	ND	7q34 amp	Y	ND	ND	
2	6	Cerebellar	ND	ND	7q34 amp	Y	ND	ND	
3	6	Cerebellar	ND	ND	7q34 amp	Y	ND	ND	
4	6	Cerebellar	ND	ND	7q34 amp	Y	ND	ND	
5	6	Cerebellar	ND	ND	7q34 amp	Y	ND	ND	
6	6	Cerebellar	ND	ND	7q34 amp	Y	ND	ND	
7	6	Cerebellar	ND	ND	7q34 amp	Y	ND	ND	
8	6	Cerebellar	ND	ND	7q34 amp	Y	ND	ND	
9	4	Cerebellar	A	A	7q34 amp	Y	Pos	WT	
10	4	Cerebellar	A	A	7q34 amp	Y	Pos	WT	
11	4	Cerebellar	N	N	N	N	Pos	WT	
12	4	Cerebellar	A	A	7q34 amp	Y	Pos	WT	
13	4	Cerebellar	N	N	ND	N	Pos	ND	
14	4	Cerebellar	A	A	7q34 amp	Y	Pos	WT	
15	4	Cerebellar	ND	ND	N	N	Pos	ND	
16	4	Cerebellar	A	A	7q34 amp	Y	Pos	WT	
17	3	Cerebellar	N	N	N	N	Pos	WT	
18	6	Cerebellar	N	N	N	N	Pos	BRAFV600E	
19	11	Cerebellar	A	A	7q34 amp	Y	Pos	WT	
20	11	Cerebellar	A	A	7q34 amp	Y	Pos	WT	
21	6	Cerebellar	A	A	ND	Y	Pos	WT	
22	11	Cerebellar	A	A	ND	Y	Pos	WT	
23	9	Cerebellar	N	N	ND	N	Pos	WT	
24	4	Cerebellar	A	A	ND	Y	Pos	WT	
25	8	Cerebellar	A	A	ND	Y	Pos	WT	
26	2	Cerebellar	A	A	ND	Y	Pos	WT	
27	9	Cerebellar	A	A	ND	Y	Pos	WT	
28	16	Cerebellar	A	A	ND	Y	Pos	WT	
29	2	Cerebellar	A	A	ND	Y	Pos	WT	
30	14	Cerebellar	A	A	ND	Y	Pos	WT	*N*=24/30
									
*Brainstem, hypothalamus and optic pathway (OP) JPA (*n*=16)*									
31	18	Brainstem	N	N	ND	N	Pos	ND	
32	6	Brainstem	N	N	ND	N	Pos	ND	
33	3	Brainstem	N	N	ND	N	Pos	ND	
34	1	Brainstem	A	A	7q34 amp	Y	Pos	WT	
35	9	Brainstem	A	A	7q34 amp	Y	Pos	WT	
36	4	Brainstem	A	A	7q34 amp	Y	Pos	WT	
37	6	Brainstem	A	N	ND	Y-HIPK2	Pos	WT	
38	6	OP	A	A	ND	Y	Pos	ND	
39	12	OP	A	A	ND	Y	Pos	ND	
40	11	OP	A	A	ND	Y	Pos	WT	
41	6	OP	ND	ND	N	N	ND	ND	
42	6	OP	ND	ND	7q34 amp	Y	ND	ND	
43	3	OP	A	A	ND	Y	Pos	WT	
44	7	OP	A	A	7q34 amp	Y	Pos	ND	
45	6	OP	ND	ND	N	N	ND	ND	
46	6	OP	ND	ND	N	N	ND	ND	*N*=10/16
									
*Hemispheric JPA (*n*=7)*									
47	6	Parietal	ND	ND	N	N	ND	ND	
48	10	Parietal	N	N	N	N	Pos	WT	
49	13	Occipital lobe	N	N	N	N	Pos	WT	
50	6	Temporal	A	N	ND	Y-HIPK2	Pos	WT	
51	4	Occipital	N	N	ND	N	Pos	WT	
52	6	Temporal	ND	ND	N	N	ND	ND	
53	15	Ventricular	ND	N	N	N	Pos	ND	*N*=1/7
									*N*=35/53

N=negative; A=amplified; ND=not done; WT=wild type; Pos=positive; qPCR=quantitative real-time PCR; pERK=phospho-ERK; IHC=immunohistochemistry.

**Table 2a:** Characteristics of the other low-grade gliomas included in this study

**Patients**	**Age (years)**	**Location**	**Pathology**	**HIP2K (qPCR)**	**BRAF (qPCR)**	**SNP array**	**Amplification of 7q34**	**pERK (IHC)**	**BRAF V600E**
*Optic pathway NF-1-associated JPA (*n*=4)*									
54	7	Optic pathway	JPA/NF1	ND	ND	N	N	ND	ND
55	7	Optic pathway	JPA/NF1	ND	ND	N	N	ND	ND
56	7	Optic pathway	JPA/NF1	ND	ND	N	N	ND	ND
57	7	Optic pathway	JPA/NF1	ND	ND	N	N	ND	ND
									
*Other low grade gliomas (*n*=33)*									
58	6	Parietal lobe	Diffuse astrocytoma	ND	ND	N	N	ND	ND
59	7	Posterior fossa	Diffuse astrocytoma	ND	ND	N	N	ND	ND
60	2	Posterior fossa	Diffuse astrocytoma	N	N	ND	N	Pos	ND
61	14	Temporal lobe	Diffuse astrocytoma	N	N	ND	N	Pos	ND
62	10	Temporal lobe	Diffuse astrocytoma	N	N	ND	N	Pos	ND
63	0.25	Temporal lobe	Diffuse astrocytoma	A	N	ND	Y-HIP2K	Pos	WT
64	6	Posterior fossa	Diffuse astrocytoma	ND	ND	N	N	ND	ND
65	13	Posterior fossa	Diffuse astrocytoma	N	N	ND	N	ND	ND
66	6	Temporal lobe	Diffuse astrocytoma	N	N	ND	N	ND	ND
67	5	Fourth ventricule	Diffuse astrocytoma	N	N	ND	N	ND	ND
68	2	Parietal lobe	Diffuse astrocytoma	N	N	ND	N	ND	ND
69	14	Posterior fossa	Diffuse astrocytoma	N	N	ND	N	ND	ND
70	12	Posterior fossa	Diffuse astrocytoma	N	N	ND	N	ND	ND
71	11	Temporal lobe	Diffuse astrocytoma	N	N	ND	N	ND	ND
72	10	Temporal lobe	Diffuse astrocytoma	N	N	ND	N	ND	ND
73	7	Temporal lobe	Diffuse astrocytoma	N	N	ND	N	ND	ND
74	9	Posterior fossa	Diffuse astrocytoma	N	N	ND	N	ND	ND
75	12	Parietal lobe	Diffuse astrocytoma	N	N	ND	N	ND	ND
76	15	Temporal lobe	Diffuse astrocytoma	N	N	ND	N	ND	ND
77	2	Posterior fossa	Diffuse astrocytoma	N	N	ND	N	ND	ND
78	9	Parietal lobe	Diffuse astrocytoma	N	N	ND	N	ND	ND
79	18	Temporal lobe	Diffuse astrocytoma	N	N	ND	N	ND	ND
80	5	Brainstem	Diffuse astrocytoma	N	N	ND	N	ND	ND
81	3	Brainstem	Diffuse astrocytoma	N	N	ND	N	ND	ND
82	2	Hippocampus	Diffuse astrocytoma	N	N	ND	N	ND	ND
83	11	Hipothalamus	Diffuse astrocytoma	N	N	ND	N	ND	ND
84	14	Posterior fossa	Diffuse astrocytoma	N	N	ND	N	ND	ND
85	6	Frontal lobe	Ganglioglioma	ND	ND	N	N	ND	ND
86	6	Brainstem	Ganglioglioma	ND	ND	N	N	ND	ND
87	3	Temporal lobe	Ganglioglioma	N	N	N	N	Pos	WT
88	16	Hippocampus	Ganglioglioma	N	A	N	N	Pos	WT
89	1	Hippocampus	Ganglioglioma	N	N	N	N	Pos	M
90	18	Cauda equina	Ependymoma	N	N	N	N	Pos	WT

JPA=juvenile pilocytic astrocytoma; M=V600E mutation; NF1=neurofibromatosis 1; SNP=single nucleotide polymorphism; qPCR=quantitative real-time PCR; pERK=phospho-ERK; IHC=immunohistochemistry; N=negative; A=amplified; ND=not done; WT=wild type; Pos=positive; M=mutated.

**Table 2b:** Clinical characteristics of samples from children with high-rade astrocytomas included in this study

Number of patients	25
	
*Gender*	
Male	14
Female	11
	
Median age	12 years (10–18 months)
	
*WHO classification*	
Astrocytomas grade IV	18
Astrocytoma grade III	7
	
*Tumor site*	
Supratentorial	18
Infratentorial	6
Mixed	1

**Table 3:** Genomic alterations in 10 JPA with no 7q34 duplications and 5 grade II astrocytomas

**Minimal common region**					**Cerebellar**	**Brainstem and optic**	**Hemispheric**	**Grade II**	
**Chr**	**Start**	**End**	**Nb SNP**	**Size (kb)**	**JPA**	**pathway JPA**	**JPA**	**astrocytoma**	**Genes**
*Homozygous deletions*									
1p36.13	17085956	17155012	11	69056	4	1	1		LOC100129182, TRNAN2, CROCC
1q21.2	147282617	147427061	15	144444	2				FJL12528, ECM1, FJ13544, TSRC1, MCL1, ENSA
2p16.2	52995459	53070421	10			1			LOC402072
2q31.2	180123158	180129913	11	6755	1		1		ZNF385B
2q13	110201336	111021091	48	819755	1	1			Mall, NPHP1, FLJ, RGPD6, RGPD7
3p21.3	37954886	37961253	15	6367	1	2			CTDSPL
3p21.1	53003023	53021256	13	18233	1	1			SFMBT1
3q28	191217916	191221750	11	3835	3	2			CCD50
4q13.2	69064675	69163188	36	98513	1	1	1		UGT2B29P, UGT2B17, LOC100132651
4q34.1	173218118	173236491	105	18373	2				GALNT17
5p15.33	812485	873185	17	60700	1	2			ZDHHC11B, ZDHHC11
5q15	97053242	97121798		8	1				LOC391813
5q35.3	180307066	180363775	11	56709	1		1		BTNL8, LOC, BTNL3
7q11.22	67054161	67482237	54			1			LOC441249
8p23.1	12257261	12388979	16	131719		2			ZNF705C, FAM, FAM,
9p11.2	44683090	44770712	20	87623	2	1	1		LOC
11q11	55124465	55209499	49	85035	1	1			OR4C11, OR4P4, OR4S2, OR
11q11	55447435	55484857	8			1			OR5I1, OR10AF1P, OR10AK1P
12p13.31	9526879	9607393	15	80515	3	2			Ovostatin
17p11.2	18292126	18398047	9	105922	1	1			NOS2B, FAM, LOC
19q13.2	46047894	46075668	22	27774	2				CYP2A6, CYP2A7
19q13.41	56230373	56263967		8		1			KLK13
20q13.2	52080333	52088118	23	7786	2				BCAS1
21q21.3	26136162	26176988		8	1				APP
									
*Chromosomal amplifications*									
1p31.1	74447282	74631475	16		1				TNNI3K
1q43	234665731	234752676	84	86946	2				EDARADD, ENO1P, LGALS8
2p11.2	86152633	86343699	20			1			POLR1A, FLJ20758, IMMT, MRPL35
4p12	47678100	47893952	50	139606	1				NPAL1, TXK, TEC
4q12	54076102	55611307	88		1				FIP1L1, LNX1, CHIC2, GSH-2, PDGFRa, KIT
5q35.3	178337857	178528476	30		1				GRM6, ZNF354C, LOC645944, ADAMTS2
5q11.2	53507913	53670668	128	162756	1				ARL15
6q27	168091860	168319676	209	227817	2	1			MLLT4, LOC100128124, KIF25, FRMD1
8p12-8p11.23	38405382	38873687	41					2	FGFR1, FLJ43582, TACC1
8p23.1	8102456	8182850	26	80395		1			FLJ10661, LOC1001
10q11.22	47007374	47167032	82	74264	2	1			LOC340844, LOC728684, ANTXRL
10q26.3	135116379	135202003	14					2	Sprn, OR6LP2, LOC399832, OR7M1P
12p13.31	7895025	8014573	56	119549	1				SLC2A14, LOC, NANOGP1, SLC2A3, NECAP1
12p11.21	31157554	31300846	55	143293	3	2	2	2	LOC100132881, OVOS2, LOC441632
12q14.2	62294703	62415375	7		1				FLJ32949, LOC390338
14q11.2	19283777	19493705	28	209929		1			All olfactory receptors
14q24.1-14q24.2	69202898	69470896	32			1			KIAA0247, SFRS5, SLC10A1, RPL7AP6, SMOC1
15q11.2	19359417	19523964	26	164548			1		LOC, OR11J2P, OR11J5P
15q26.3	99310512	99791921	204	481410	1				LRRK1, CHSY1, SELS, SNRPA1, PCSK6
17q25.3	74877129	74905197	48	28069		1			LOC, HRNBP3
19q13.31	47948855	48150403	26	201549	2				Pregnancy specific beta-1-glycoproteins
19q13.42	59971240	60054671	14	83432	1	3	2		KIR2DL1-4, KIR3DP1, KIR3DL1, KIR2DS1-4
20p13	1593502	1663448	41	69947	1				LOC
21q22.2	39823301	39835430	15	12130	1				LOC
22q11.23	23991725	24240667	94	248943	1				IGLL3, LRP5L, LOC, CRYBB2P1
22q11.21	17257787	17388108	73	130322	1				LOC, DGCR6, PRODH, DGCR5
