# Supplementary material for: Duplication of 7q34 is specific to juvenile pilocytic astrocytomas and a hallmark of cerebellar and optic pathway tumours
Source: Br J Cancer. 2009 Jul 14;101(4):722–33. doi: 10.1038/sj.bjc.6605179 (PMC2736806; doi:10.1038/sj.bjc.6605179)
Supplement: Supplementary Table 1 [file 6605179x1.pdf]

Supplementary Table 1

Primers used for real-time quantitative PCR:

BRAF exon 12:
F: 5’ AGCACCTACACCTCAGCAGTTACA 3’
R: 5’ ACCACTGGGAACCAGGAGCTAATA 3’
HIPK2:
F: 5' CTGGTGGGGTAGCATCTTCT 3'
R: 5' TTGCTGGAGGACTTGGACTT 3'
TBXAS1:
F: 5’ TTTCCTGCCTGTTGCTCACACTCT 3’
R: 5’ TCCAAGACGCTGCCAATCCAGTTA 3’
MRPS33:
F: 5’TTCCGCTGCCGTAATTTCCTCTCA 3’
R: 5’TTAATCTGCAGCCCACCACCCAAA 3’
LINE-1
F: 5’AAAGCCGCTCAACTACATGG 3’
R: 5’TGCTTTGAATGCGTCCCAGAG 3’

Mutational analysis for *BRAF*
BRAF exon 12:
F: 5’AAGGGGATCTCTTCCTGTATCC 3’
R: 5’ GAGTCCCGACTGCTGTGAAC 3’

BRAF exon 15:
F: 5’ TTGACTCTAAGAGGAAAGATGAAGT 3’
R: 5’TTGAGACCTTCAATGACTTTCTAGT 3’
