# Supplementary material for: Duplication of 7q34 is specific to juvenile pilocytic astrocytomas and a hallmark of cerebellar and optic pathway tumours
Source: Br J Cancer. 2009 Jul 14;101(4):722–33. doi: 10.1038/sj.bjc.6605179 (PMC2736806; doi:10.1038/sj.bjc.6605179)
Supplement: Supplementary Table 2 [file 6605179x2.pdf]

Deletions (CNV) detected by GqCNV and PennCNV in at least 3 tumors

	Locus (Mb)						Detected in database
Chr	Start	End	Size	Nb SNP	Nb tumors	Genes	of Genomic Variants
1p36.13	17086767	17155012	68246	11	6	3 genes LOC100129182, TRNAN2, CROCC	no
2p22.3	34544587	34592120	35482	32	5	no gene	no
2q33.3	208057503	208071307	13805	17	4	no gene	no
3q26.1	163746526	164238477	491952	105	5	no gene	no
3q28	192547222	192555922	8701	18	5	1 gene CCD50	no
4q13.1	64350537	64392223	41687	18	4	no gene	no
5q23.1	117414333	117421055	6723	13	4	no gene	no
6p21.33-6p21.32	29965944	32763196	37147	123	10	168 genes	no
6q14.1	79036117	79086086	49970	46	6	no gene	no
7q34	141419097	141439888	20792	21	5	1 MGAM	no
8p11.23-8p11.22	39350791	39509376	158586	38	12	2 genes ADAM5P, ADAM3A	no
9p11.2	44683090	44797463	114374	20	4	1 gene LOC100131427	no
12p13	9526879	9607393	80515	15	5	1 OVOS	no
12p11.22	30125165	30134898	9734	10	4	no gene	no

Amplifications (CNV) detected by GqCVN and PennCNV in at least 3 tumors

	Locus (Mb)						Detected in database
Chr	Start	End	Size	Nb SNP	Nb tumors	Genes	of Genomic Variants
6q27	168091860	168319676	227817	209	3	4 genes MLLT4, LOC100128124, KIF25, FRMD1	yes
**7q34**	**139501685**	**140079362**	**577678**	**124**	**20**	**region of interest**	**NO**
10q11.22	47007374	47167032	74264	82	3	3 genes LOC340844, LOC728684, ANTXRL	yes
12p11.21	31157554	31300846	143293	55	9	3 genes LOC100132881, OVOS2, LOC441632	no
19q13.42	59971240	60054671	83432	14	6	6 genes KIR2DL1-4, KIR3DP1, KIR3DL1, KIR2DS1-4	no

Supplementary Table 2: Genomic alterations in the data set generated on the Hap300-Duo and 610-Qad human arrays detected by both GqCNV and PennCNV algo

rithms.
