# Supplementary material for: Duplication of 7q34 is specific to juvenile pilocytic astrocytomas and a hallmark of cerebellar and optic pathway tumours
Source: Br J Cancer. 2009 Jul 14;101(4):722–33. doi: 10.1038/sj.bjc.6605179 (PMC2736806; doi:10.1038/sj.bjc.6605179)
Supplement: Supplementary Table 3 [file 6605179x3.pdf]

Supplementary Table 3: Results *BRAF* sequencing for exon 11 (BRAFE11) and exon 15 (BRAFE15)

**PCR primer pair**	**BRAFE11**	**BRAFE15**
**PCR size (*bp*)**	426	354
**SNP position in contig**	Contig1: 319	Contig2: 173
**Genotype (*Wildtype/Mutant*)**	T/A	T/A
**Position in UCSC Genome Browser (*March 20***	Chr7: 140127723	Chr7: 140099605
**Position in Intron/Exon/UTR**	Intron	Exon
**Function (*Wiltype vs Mutant*)**	N/A	Val (GTG) vs Glu (GAG)
**UCSC SNP reference number (*March 2006*)**	New SNP	New SNP
**Sample number as per Tables 1 and 2**		
18	TT	**TA**
9	TT	TT
10	TT	TT
11	TT	TT
12	TT	TT
36	TT	TT
50	TT	TT
14	TT	TT
16	TT	Missing Sequence
17	TT	TT
34	TT	TT
35	TT	TT
36	TT	TT
19	TT	TT
20	TT	TT
53	TT	TT
21	TT	TT
72	TT	TT
22	TT	TT
63	TT	TT
67	TT	TT
68	TT	TT
69	TT	**TA**
23	TT	TT
47	TT	TT
24	TT	TT
30	TT	TT
25	TT	TT
55	TT	TT
26	TT	TT
27	TT	TT
54	TT	TT
28	TT	TT
29	TA	TT
75	TT	TT
37	TT	TT
70	TT	TT
73	TT	TT
71	TT	TT
74	TT	TT
**Surrounding Sequence**	gttcgcatcaataaactattttgat**T**agttt caggactcctccaaaagttt	ataggtgattttggtctagctacag**T**gaa atctcgatggagtgggtccca
